# Multiple Events of Allopolyploidy in the Evolution of the Racemose Lineages in *Prunus* (Rosaceae) Based on Integrated Evidence from Nuclear and Plastid Data

**DOI:** 10.1371/journal.pone.0157123

**Published:** 2016-06-13

**Authors:** Liang Zhao, Xi-Wang Jiang, Yun-juan Zuo, Xiao-Lin Liu, Siew-Wai Chin, Rosemarie Haberle, Daniel Potter, Zhao-Yang Chang, Jun Wen

**Affiliations:** 1 College of Life Sciences, Northwest A&F University, Yangling, Shaanxi, 712100, China; 2 College of Life Sciences, Jianghan University, Wuhan, Hubei, 430056, China; 3 Shanghai Chenshan Plant Science Research Center, Chinese Academy of Sciences / Shanghai Chenshan Botanical Garden, 3888 Chenhua Road, Songjiang, Shanghai, 201602, China; 4 School of Applied Chemistry and Biological Engineering, Weifang Engineering Vocational College, Qingzhou, Shandong, 262500, China; 5 Department of Plant Sciences, MS2, University of California Davis, Davis, California, 95616, United States of America; 6 Department of Biology, Pacific Lutheran University, Tacoma, Washington, 98447, United States of America; 7 Department of Botany, National Museum of Natural History, MRC 166, Smithsonian Institution, Washington, DC, 20013–7012, United States of America; Saint Mary's University, CANADA

## Abstract

*Prunus* is an economically important genus well-known for cherries, plums, almonds, and peaches. The genus can be divided into three major groups based on inflorescence structure and ploidy levels: (1) the diploid solitary-flower group (subg. *Prunus*, *Amygdalus* and *Emplectocladus*); (2) the diploid corymbose group (subg. *Cerasus*); and (3) the polyploid racemose group (subg. *Padus*, subg. *Laurocerasus*, and the *Maddenia* group). The plastid phylogeny suggests three major clades within *Prunus*: *Prunus-Amygdalus-Emplectocladus*, *Cerasus*, and *Laurocerasus-Padus-Maddenia*, while nuclear ITS trees resolve *Laurocerasus-Padus-Maddenia* as a paraphyletic group. In this study, we employed sequences of the nuclear loci *At103*, ITS and *s6pdh* to explore the origins and evolution of the racemose group. Two copies of the *At103* gene were identified in *Prunus*. One copy is found in *Prunus* species with solitary and corymbose inflorescences as well as those with racemose inflorescences, while the second copy (II) is present only in taxa with racemose inflorescences. The copy I sequences suggest that all racemose species form a paraphyletic group composed of four clades, each of which is definable by morphology and geography. The tree from the combined *At103* and ITS sequences and the tree based on the single gene *s6pdh* had similar general topologies to the tree based on the copy I sequences of *At103*, with the combined *At103-*ITS tree showing stronger support in most clades. The nuclear *At103*, ITS and *s6pdh* data in conjunction with the plastid data are consistent with the hypothesis that multiple independent allopolyploidy events contributed to the origins of the racemose group. A widespread species or lineage may have served as the maternal parent for multiple hybridizations involving several paternal lineages. This hypothesis of the complex evolutionary history of the racemose group in *Prunus* reflects a major step forward in our understanding of diversification of the genus and has important implications for the interpretation of its phylogeny, evolution, and classification.

## Introduction

*Prunus* L. belongs to subfamily Amygdaloideae of the family Rosaceae [1]. It consists of ca. 250–400 species of deciduous and evergreen trees and shrubs widely distributed in the temperate zone of the Northern Hemisphere and in the subtropics and tropics of both the New and Old Worlds [2–4]. *Prunus* is economically important as the source of many temperate fruit and nut crops, such as almonds, cherries, plums, and peaches, as well as for timber and ornamentals [5]. The genus is defined based on a combination of characters including presence of leaf glands, a solitary carpel, superior ovary position, fruit a drupe, and solid pith [2, 6].

The taxonomy of *Prunus* has been controversial, especially concerning the generic delimitation and infrageneric classification [2, 5, 7]. The most widely accepted classification of this genus consists of five subgenera: *Prunus*, *Amygdalus* (L.) Focke, *Cerasus* Pers., *Laurocerasus* Koehne, and *Padus* (Moench) Koehne [2]. However, some treatments segregated the group into multiple genera [8, 9]. Recent phylogenetic studies support a broad circumscription of *Prunus* [1, 3, 4, 10]. The most recent classification of the genus recognized only three subgenera: *Prunus*, *Cerasus*, and *Padus*, with a broader concept of subgenus *Padus* that included *Laurocerasus* and the former genera *Maddenia* Hook. f. & Thoms and *Pygeum* Gaertn. [7].

Several molecular phylogenetic studies [3–6, 10, 11–15] have been conducted to investigate the evolutionary relationships of *Prunus* using both plastid (*rbcL*, *matK*, *ndhF*, *rps16*, *rpl16*, *trnL-L-F*, and *trnS-S-G*) and nuclear (nrITS and *s6pdh*) sequences. All previous studies have clearly supported the monophyly of *Prunus* s.l. The plastid data have supported three main clades within *Prunus*, which correspond to three groups that can be identified based on inflorescence structure [4]: (1) the deciduous solitary-flower group, including subg. *Prunus*, *Amygdalus*, and *Emplectocladus*; (2) the deciduous corymbose inflorescence group, referring to subg. *Cerasus*; and (3) the racemose inflorescence group, containing subg. *Laurocerasus*, comprising evergreen species (also including southeast Asian species formerly assigned to the genus *Pygeum* [16]), as well as subg. *Padus* and the former genus *Maddenia*, both comprising deciduous species with temperate distributions [4, 6, 17]. Most taxa of the solitary-flower and corymbose groups are diploid (2*n* = 2*x* = 16), while taxa of the racemose group usually have higher ploidy levels with 2*n* = 4*x* = 32 or sometimes 2*n* = 8*x* = 64 [4, 11, 16–19].

**Fig 1:**
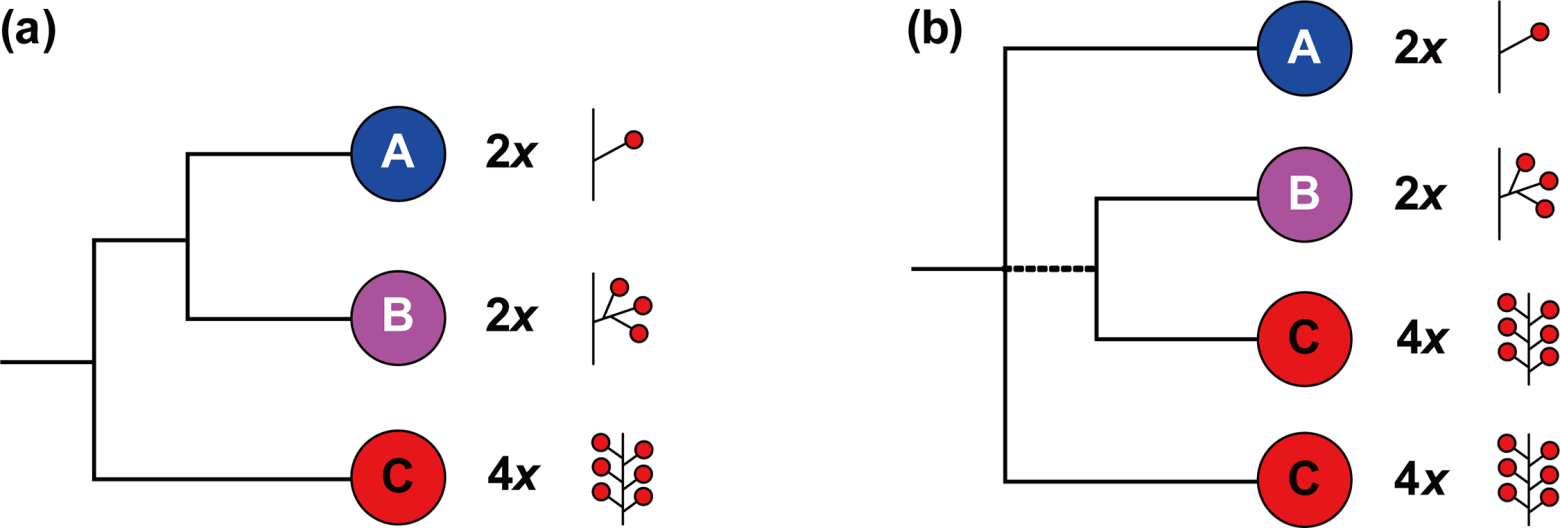
**Summary of phylogenetic relationships in *Prunus*, based on the plastid DNA data (a), and nuclear sequences (b)** (simplified from Chin et al.[4]). A = solitary inflorescence clade, including subgenus *Prunus* and *Amygdalus*; B = corymbose inflorescence clade, which refers to subgenus *Cerasus*; C = racemose inflorescence group, including subgenus *Laurocerasus*, *Padus*, *Maddenia* and *Pygeum* group.

Plastid phylogenies support the monophyly of the three groups described above and resolve the first two clades as sister to one another [4] (Fig 1A). In contrast, nrITS DNA data have supported a different topology, with one clade consisting of members of subgenera *Prunus*, *Amygdalus*, and *Emplectocladus*, identical to clade A in the plastid phylogeny (the solitary flower group), a second clade including most members of *Cerasus* (the corymbose inflorescence group, clade B in the plastid phylogeny), and species of *Laurocerasus*, *Padus* and *Maddenia* (the racemose inflorescence group) comprising a paraphyletic group of lineages (Fig 1B; also see Fig 4 in Chin et al. [4]), rather than a clade as in the plastid phylogeny (clade C).

The incongruences in relationships among the racemose inflorescence lineages resolved in the maternally inherited plastid phylogeny vs. in the biparentally inherited nuclear ITS phylogeny have led to the suggestion of a hybrid origin of this group [4]. However, a number of molecular genetic processes (e.g., ancient or recent array duplication events, genomic harboring of pseudogenes in various states of decay, and/or incomplete intra- or interarray homogenization) can impact ITS sequences in ways that may mislead phylogenetic inference [20]. Thus, more nuclear markers, especially low-copy nuclear genes that can track both parents’ genomes in hybrids [21], are needed to test the hypothesis of the allopolyploid origin of racemose *Prunus* [4].

This study aims to provide further insights into the phylogenetic relationships within *Prunus* using sequences of the low-copy nuclear *At103* [22, 23] and *s6pdh* [24] genes, as well as data from ITS. The primary goal of this study is to clarify the evolution of the polyploid racemose group using these nuclear sequences, integrating evidence from the established plastid phylogeny.

## Materials and Methods

### Ethics Statement

Species of *Prunus*, *Prinsepia*, *Physocarpus* and *Oemleria* sampled in this study do not represent endangered or protected plants. Thus no specific permits were required for the collection of samples, which complied with all relevant regulations. The species information is provided in Tables 1–3. All voucher specimens are deposited in the US National Herbarium (US) or the Herbarium of University of California, Davis (DAV).

**Table 1:** Taxa and *At103* gene GenBank accession numbers of *Prunus* and outgroups sampled for this study. All voucher specimens (except one collection *Potter 081118* deposited in DAV) are deposited in the US National Herbarium (US).

Taxon	Voucher	Location	Gene bank Accession number
**Subgenus. 1. *Prunus* L.**			
*P*. *armeniaca* L.	*Wen 10326*	Russia, Stavropol region, Pjatigorsk	KU525428
*P*. *armeniaca* L.	*Wen 11933*	USA, Virginia, Fairfax	KU525429
*P*. *divaricata* Ledeb.	*Wen 10350*	Russia, Karachaevo—Cherkesskaja	KU525431
*P*. *glandulosa* Thunb.	*Wen 11932*	USA, Virginia, Fairfax	KU525427
*P*. *mandshurica* (Maxim.) Koehne	*Wen 5444*	USA, Arnold Arboretum	KU525436
*P*.* mume (Siebold) Siebold & Zucc*.	*Wen 11942*	USA, Washington D.C.	KU525435
*P*.* mume (Siebold) Siebold & Zucc*.	*Wen 12030*	China, Fujian Prov., Wuyishan	KU525457
*P*. *salicina* Lindl.	*Wen 8032*	China, Gansu Prov., Xihe Co.	KU525443, KU525444
*P*. *salicina* Lindl.	*Wen 10894*	Vietnam, Lao Cai Prov., Sa Pa	KU525456
*P*. *salicina* Lindl.	*Wen 11928*	USA, Washington D.C., cult.	KU525437- KU525442; KU525445- KU525449; KU525453- KU525455; KU525451, KU525516
*P*. *sibirica* L.	*Wen 8532*	China, Beijing Shi, Baihuashan	KU525430
*P*. *murrayana* Palmer	*Wen 7283*	USA, Texas, Brewster	KU525433
*P*. *nigra* Desf.	*Wen 9904*	USA, Wisconsin, Dane Co.	KU525434
*P*. *nigra* Desf.	*Wen 11112*	USA, Florida, Wakulla Co.	KU525450, KU525452
*P*. *rivularis* Scheele	*Wen 11988*	USA, Texas, Kerr Co.	KU525426
**Subgenus. 2. *Amygdalus* (L.) Focke**			
*P*. *mira* Koehne	*Wen 9207*	China, Tibet, Tzayu Co.	KU525424
*P*. *persica* (L.) Batsch	*Wen 10883*	Vietnam, Lao Cai Prov., Sa Pa	KU525401
*P*. *tenella* Batsch	*Wen 237*	USA, Arnold Aboretum	KU525432
*P*. *triloba* Lindl.	*Wen 11935*	USA, Pensylvania, Swarthmore College, cult.	KU525425
**Subgenus. 3. *Cerasus* Pers.**			
*P*. *campanulata* Maxim.	*Wen 11239*	China, Zhejiang Prov., Qingyuan Co.	KU525406
*P*. *campanulata* (Maxim.) Yü et Li	*Wen 12040*	China, Fujian Prov., Wuyishan	KU525459
*P*. *cerasoides* Buch. -Ham. ex D. Don	*Wen 10126*	Indonesia, Java, cult.	KU525415
*P*. *cerasoides* Buch. -Ham. ex D. Don	*Wen 10833*	Vietnam, Lao Cai Prov., Sa Pa	KU525405
*P*. *cerasoides* Buch. -Ham. ex D. Don	*Tibet 3163*	China, Tibet	KU525402
*P*. *clarofolia* Schneid.	*Tibet 2839*	China, Tibet	KU525416
*P*. *clarofolia* Schneid.	*Wen 9211*	China, Tibet, Bomi Co.	KU525417
*P*. *dielsiana* (Schneid.) Yü et Li	*Wen 9295*	China, Hunan Prov., Xinning Co., cult.	KU525414, KU525460
*P*. *discoidea* (Yu & Li) Wei & Chang	*Wen 11284*	China, Zhejiang Prov., Linan	KU525413
*P*. *maackii* Rupr.	*Wen 11794*	USA, Washington DC	KU525400
*P*. *maackii* Rupr.	*Wen 5405*	China, Jilin Prov., Tonghua	KU525458
*P*. *mahaleb* L.	*Wen 1372*	USA, Arnold Arboretum	KU525518
*P*. *mahaleb* L.	*Wen 355*	USA, Arnold Aboretum, cult.	KU525399
*P*. *nipponica* Matsum.	*Wen 11797*	USA, Washington DC	KU525409
*P*. *serrula* Franch.	*Wen 9155*	China, Tibet, Gongbujiangda Co.	KU525404
*P*. *trichostoma* (Koehne) Yü et Li	*Wen 12086*	China, Sichuan Prov., Emeishan	KU525407
*P*. *subhirtella* Miq.	*Wen 11795*	USA, Washington DC	KU525408
*P*. *takesimensis* Nakai	*Wen 11937*	USA, Pensylvania, Swarthmore College	KU525412
*P*. *tomentosa* Thunb	*Wen 8091*	China, Chongqing Shi, Chengkou Co.	KU525403

**Table 2:** Taxa and *At103* gene GenBank accession numbers of *Prunus* and outgroups sampled for this study. All voucher specimens (except one collection *Potter 081118* deposited in DAV) are deposited in the US National Herbarium (US).

Taxon	Voucher	Location	Gene bank Accession number
*P*. *tomentosa* Thunb	*Wen 11210*	China, Zhejiang Prov., Longquan Co.	KU525411
*P*. *trichostoma* Koehne	*Wen 12141*	China, Sichuan Prov., Emei	KU525410
*P*. *yedoensis* Matsum.	*Wen 11796*	USA, Washington DC	KU525517
**Subgenus. 4. *Padus* (Moench) Koehne**			
*P*. *alabamensis* Mohr.	*Alvin s*.*n*.	USA, Alabama	KU525374-KU525376; KU525483- KU525485;
*P*. *buergeriana* Miq.	*Wen 11290*	China, Zhejiang Prov., Anji Co.	KU525502
*P*. *napaulensis* (Ser.) Steud.	*Wen 9217*	China, Tibet, Bomi Co.	KU525362-KU525370; KU525503-KU525505; KU525519
*P*. *padus* L.	*Wen 10319*	Russia, Moscow, Serpukhov	KU525377-KU525381; KU525495-KU525501;
*P*. *wilsonii* (Schneid.) Koehne	*Wen 11174*	China, Zhejiang Prov., cult.	KU525418-KU525423; KU525490-KU525494
**Subgenus. 5. *Laurocerasus* Koehne**			
*P*.* integrifolia (Sudw*.*) Sarg*.	*Wen 8620*	Peru, Oxapampa	KU525463
*P*. *fordiana* Dunn	*Wen 10845*	Vietnan, LaoCai Prov., Sa Pa	KU525512
*P*. *laurocerasus* L.	*Wen 10366*	Russian Federation	KU525348, KU525506
*P*. *tucumanensis* Lillo	*Nee & Wen 53882*	Bolivia	KU525488
*P*. *wallichii* Steud.	*Wen 10877*	Vietnan, LaoCai Prov., Sa Pa	KU525371-KU525373; KU525486-KU525487;
***Pygeum* group**			
*P*. *africana* (Hook. f.) Kalkman	*Wen 9688*	Madagascar, Toamsina	KU525361
*P*. *arborea* (Blume) Kalkman	*Wen 10927*	Vietnam, Ninh Binh Prov.	KU525359-KU525360; KU525465-KU525469;
*P*. *arborea* (Blume) Kalkman	*Wen 10246*	Indonesia, Sulawesi Tenggara, Sanggona	KU525358, KU525514 KU525472-KU525476
*P*. *costata* (Hemsl.) Kalkman	*Wen 10106*	Indonesia, Java., cult.	KU525356
*P*. *grisea* (Blume ex Müll. Berol.) Kalkman	*Wen 11037*	Vietnam, Lam Dong Prov.	KU525349-KU525354;
*P*. *grisea* (Blume ex Müll. Berol.) Kalkman var. *microphylla* Kalkman	*Potter 081118* (DAV)	Indonesia, West Papua Prov., Manokwari District	KU525355, KU525357
*P*. *lancilimba* (Merr.) Kalkman	*Wen 10851*	Vietnan, LaoCai Prov., Sa Pa	KU525470-KU525471; KU525477-KU525482
*P*. *lancilimba* (Merr.) Kalkman	*Wen 10829*	Vietnan, LaoCai Prov., Sa Pa	KU525464
***Maddenia* group**			
*P*. *himalayana* Wen	*Tibet 2612*	China, Tibet	KU525382-KU525384; KU525386-KU525388; KU525390-KU525398; KU525508-KU525509 KU525515, KU525520
*P*. *hypoleuca* (Koehne) Wen	*Wen 665*	USA, Arnold Arboretum, cult.	KU525385, KU525389, KU525489, KU525507, KU525510
**Outgroups**			
*Physocarpus opulifolius* (L.) Maxim.	*Wen 11943*	USA, Virginia, Fairfax Co., cult.	KU525461
*Prinsepia utilis* Royle	*Wen 9174*	China, Tibet, Linzhi	KU525462

**Table 3:** Taxa and *At103* and ITS GenBank accession numbers of *Prunus* and outgroups sampled for this study. All voucher specimens are deposited in the US National Herbarium (US). Newly generated sequences are indicated by an asterisk (*).

Taxon	Voucher	Location	GeneBank Accession (ITS/At103)
**Subgenus. 1. *Prunus* L.**			
*P*. *armeniaca* L.	*Wen 10326*	Russia, Stavropol region, Pjatigorsk	JQ776883; KU525428
*P*. *divaricata* Ledeb.	*Wen 10350*	Russia, Karachaevo—Cherkesskaja	JQ776885; KU525431
*P*. *mandshurica* (Maxim.) Koehne	*Wen 5444*	USA, Arnold Arboretum	JQ776884; KU525436
*P*. *salicina* Lindl.	*Wen 10894*	Vietnam, Lao Cai Prov., Sa Pa	JQ776887; KU525456
**Subgenus. 2. *Amygdalus* (L.) Focke**			
*P*. *persica* (L.) Batsch	*Wen 10883*	Vietnam, Lao Cai Prov., Sa Pa	JQ776821; KU525401
**Subgenus. 3. *Cerasus* Pers.**			
*P*. *discoidea* (Yu & Li) Wei & Chang	*Wen 11284*	China, Zhejiang Prov., Linan	JQ776825; KU525413
*P*. *nipponica* Matsum.	*Wen 11797*	USA, Washington DC	JQ776829; KU525409
*P*. *maackii* Rupr.	*Wen 11794*	USA, Washington DC	JQ776862; KU525400
*P*. *yedoensis* Matsum.	*Wen 11796*	USA, Washington DC	JQ776833; KU525517
*P*. *mahaleb* L.	*Wen 1372*	USA, Arnold Aboretum	JQ776828; KU525518
**Subgenus. 4. *Padus* (Moench) Koehne**			
*P*. *padus* L.	*Wen 10319*	Russia, Moscow, Serpukhov	KX013510*; KU525380
*P*. *buergeriana* Miq.	*Wen 11290*	China, Zhejiang Prov., Anji Co.	JQ776852; KU525502
*P*. *wilsonii* (Schneid.) Koehne	*Wen 11174*	China, Zhejiang Prov., cult.	JQ776881; KU525492
**Subgenus. 5. *Laurocerasus* Koehne**			
*P*. *laurocerasus* L.	*Wen 10366*	Russian Federation	JQ034155; KU525348
*P*. *tucumanensis* Lillo	*Nee & Wen 53882*	Bolivia	KX013511*; KU525488
***Pygeum* group**			
*P*. *africana* (Hook. f.) Kalkman	*Wen 9688*	Madagascar, Toamsina	KX013504*; KU525361
*P*. *arborea* (Blume) Kalkman	*Wen 10927*	Vietnam, Ninh Binh Prov.	KX013505*; KU525466
*P*. *costata* (Hemsl.) Kalkman	*Wen 10106*	Indonesia, Java., cult.	KX013506*; KU525356
*P*. *grisea* (Blume ex Müll. Berol.) Kalkman	*Wen 11037*	Vietnam, Lam Dong Prov.	KX013507*; KU525351
*P*. *lancilimba* (Merr.) Kalkman	*Wen 10829*	Vietnan, LaoCai Prov., Sa Pa	KX013509*; KU525464
*Maddenia group*			
*P*. *himalayana* Wen	*Tibet 2612*	China, Tibet	KX013508*; KU525508
*P*. *hypoleuca* (Koehne) Wen	*Wen 665*	USA, Arnold Aboretum, cult.	JQ776888; KU525510
**Outgroup**			
*Physocarpus opulifolius* (L.) Maxim.	*Wen 11943*	USA, Virginia, Fairfax Co., cult.	JQ34169; KU525461

### Taxon sampling and outgroup selection

For the *At103* gene sequences, 47 species of *Prunus* representing all five subgenera recognized by Rehder [2] were sampled (Tables 1 and 2). All samples were used in previous studies by Chin et al. [4] and Liu et al. [15]. In addition, two outgroup species, *Prinsepia utilis* and *Physocarpus opulifolius*, were selected based on previous phylogenetic studies of Rosaceae [1]. We also included ITS data for 23 of the species for which we obtained sequences of *At103* (Table 3), covering the five subgenera defined by Rehder [2]. For the *s6pdh* data, we included 26 species, also representing all five subgenera of *Prunus* recognized by Rehder [2] with *Oemleria cerasiformis* as the outgroup (Table 4). Our samples were collected throughout the range of *Prunus* including from Asia, Europe, North America, South America and Africa, and represent all three types of inflorescence structures. We especially made sure that the the racemose inflorescence group was well represented in our sampling, because this group is the most species-rich and morphologically diverse group in the genus, and the one whose phylogenetic relationships are in question and whose origins we sought to clarify.

**Table 4:** Taxa and *s6pdh* gene GenBank accession numbers of *Prunus* and outgroups sampled for this study. All voucher specimens are deposited in the US National Herbarium (US) and and the Herbarium of University of California, Davis (DAV). Newly generated sequences are indicated by an asterisk (*).

Taxon	Voucher	Location	GeneBank Accession
**Subgenus. 1. *Prunus* L.**			
*P*. *armeniaca* L.			EU056696
*P*. *cerasifera* Ehrh.			EU056697
*P*. *consociiflora* C. K. Schneid.			DPRU2457
*P*. *fremontii* S. Watson			AF414986
*P*. *salicina* Lindl.			AF414982
*P*. *subcordata* Benth.			AF414980
*P*. *mexicana* S. Watson			AF414977
**Subgenus. 2. *Amygdalus* (L.) Focke**			
*P*. *fasciculata* (Torr.) A. Gray			AF414978
*P*. *persica* (L.) Batsch			AF414988
**Subgenus. 3. *Cerasus* Pers.**			
*P*. *clarofolia* C.K.Schneid.	*Wen 9277* (US)	China, Hunan Prov., Xinning Co.	KU525529*
*P*. *emarginata* (Douglas ex Hook.) Walp.	*EB139*	USA, California, El Dorado Co.	AF504298, AF504299, AF504300, AF504301, AF504302
*P*. *emarginata* (Douglas ex Hook.) Walp.	*DPRU 2214*	USA, Davis, USDA National Clonal Germplasm Repository	AF414985, AF504303, AF504304
*P*. *fruticosa* Pall.			AF414996
**Subgenus. 4. *Padus* (Moench) Koehne**			
*P*. *padus* L.			AF415007
*P*.* serotina subsp*.* virens (Wooton & Standl*.*) McVaugh*	*Beck & Estes s*.*n*.	USA, Texas, Jeff Davis Co.	KU525524*
*P*. *virginiana* L.			AF504297
**Subgenus. 5. *Laurocerasus* Koehne**			
*P*. *brachybotrya* Zucc.	*Wen 8755* (US)	Mexico, Vera Cruz.	KU525523*, KU525531*
*P*. *caroliniana* (Mill.) Aiton			AF415009, AF455049, AF415008
*P*. *integrifolia* Walp.	*Wen 8620* (US)	Peru, Oxapampa	KU525521*, KU525522*
*P*. *ilicifolia* (Nutt. ex Hook. & Arn.) D. Dietr.			AF414974
*P*. *laurocerasus* L.			AF415010
*P*. *lusitanica* L.			AF504296
*P*. *myrtifolia* (L.) Urb.	*Vincent et al*. *12720*	Bahamas, Great Abaca Island	KU525530*
***Pygeum* group**			
*P*. *africana* (Hook. f.) Kalkman	*DPRU2557*	Kenya, Trans Nzoia	KU525525*
*P*. *henryi* (C. K. Schneid.) Koehne	*Wen 8463* (US)	China, Yunnan Prov.	KU525528*
*P*. *polystachya* (Hook. f.) Kalkman	*Wen 8407* (US)	Malaysia, Selangor	KU525526*, KU525527*
**Outgroup**			
*Oemleria cerasiformis* (Torr. & A. Gray ex Hook. & Arn.) J. W. Landon			AF415011

### DNA isolation, amplification, cloning, and sequencing

Total genomic DNA was extracted from silica gel-dried or herbarium material using the Plant DNA Extraction Kit AGP965/960 (AutoGen, Holliston, Massachusetts, U.S.A.) or the DNeasy Plant Mini Kit (Qiagen, Crawley, UK). All PCR amplifications were performed in 25-μL reactions containing 1.5 mM MgCl_2_, 0.2 mM of each dNTP, 0.4 mM of each primer, 1 U of *Taq* DNA polymerase (Qiagen), and approximately 10–50 ng of the template DNA.

The PCR primer pair for *At103* was “F” (CTTCAAGCCMAAGTTCATCTTC TA) and “R” (TTGGCAATCATTGAGGTACATNGTMACATA) as in Li et al. [23], and the amplification conditions were: 3 min initial denaturation at 95°C, 35 cycles of 30 s denaturation at 94°C, 45 s annealing at 50°C, and 60 s extension at 72°C, followed by a final extension of 5 min at 72°C.

The PCR products were cleaned with ExoSAP-IT (cat. #78201, USB Corporation, Cleveland, Ohio, U.S.A.). Purified products were sequenced with BigDye 3.1 reagents on an ABI 3730 automated sequencer (Applied Biosystems, Foster City, California, U.S.A.) from both directions. The forward and reverse sequences were assembled using Geneious v.8.1.2. (http://www.geneious.com) [25]. Special attention was paid to those sites with overlapping peaks in the chromatograms, because they may indicate intra-individual variation (polymorphisms). If an obviously overlapping signal was detected in both the forward and reverse chromatograms, the site was considered to be putatively polymorphic between alleles or copies. Those samples with polymorphic sites were cloned using the TOPO TA cloning kit (Invitrogen. Carlsbad. California, USA), following the supplied protocol. The bacterial cells picked from insert-containing colonies were directly selected as a template for PCR with the M13 forward and reverse primers. At least two clones per individual were selected and sequenced.

The nuclear ribosomal ITS regions were amplified using primers “ITS5a” (CCTTATCATTTAGAGGAAGGAG) and “ITS4” (TCCTCCGCTTATTGATATGC) as in Stanford et al. [26]. In addition, we used 15 sequences from our previously published studies [4, 11]. The PCR program was as follows: an initial 5 min at 95°C, followed by 38 cycles of 40 s at 94°C, 45 s at 52°C, and 1 min 20 s at 72°C, and a final extension cycle of 7 min at 72°C.

For the *s6pdh* sequences, because intron 1 was highly divergent and difficult to align, we only used the region from the second to the sixth exon. The *s6pdh* sequences from *Prunus consociiflora*, *P*. *serotina* subsp. *virens*, *P*. *napaulensis*, *P*. *brachypoda*, *P*. *integrifolia*, *P*. *myrtifolia*, *P*. *polystachyac*, *P*. *africana* and *P*. *integrifolia* were produced by PCR amplification with primers *s6pdh-k* “AGCTCATTACAAGAGTGA AG CAGACGTTGG”/*s6pdh-p* “AGAGTGGTCCTGGATTTCTTATCTA”, or with the primer combinations *s6pdh-k* “AGCTCATTACAAGAGTGAAGCAGACGTTG G”/*s6pdh-h* “AGACCAATGCTGCGAACTAAGCCC” and *s6pdh-c* “TTTGGAATT CAGACCATGGGCATG”/*s6pdh-p* “AGAGTGGTCCTGGATTTCTTATCTA”, which yield overlapping PCR products [12]. In addition, we used 27 sequences from previously published studies [12, 27]. The PCR amplification conditions were as follows: an initial 10 min at 95°C, followed by 35 cycles of 30 s at 95°C, 1 min at 54°C, and 2 min at 72°C, and a final extension cycle of 7 min at 72°C [12]. We also cloned sequences of *Prunus brachypoda*, *P*. *integrifolia* and *P*. *polystachya*.

The PCR products were purified using ExoSAP-IT (USB Corporation, Cleveland, Ohio, USA). Amplicons were directly sequenced in both directions using the amplification primers. Cycle sequencing reactions were conducted using the BigDye 3.1 reagents. After being cleaned up by the Sephadex columns, the sequencing products were run on an ABI 3730 automated sequencer (Applied Biosystems, Foster City, California, USA).

### Data analyses

Sequences were aligned with MUSCLE [28] and adjusted manually in Geneious v.8.1.2 [25].

For the *At103* gene, phylogenetic analyses employed 173 sequences after excluding identical sequences from the clones of the same accession. The analyses were first conducted using maximum likelihood (ML) with PhyML version 3.0 [29]. The best-fit nucleotide substitution model for the dataset was determined based on Akaike Information Criterion (AICc) in jModelTest v.2.1.7 [30, 31]. Nodal robustness on the ML tree was estimated by the nonparametric bootstrap (1000 replicates). To visualize the conflicting evolutionary signals in the *At103* data and highlight reticulate evolution, a neighbornet diagram was generated based on uncorrected-*P* distance matrix, using Splitstree 4.13.1 [32]. Bootstrap support of each group was estimated with 1000 replicates.

We combined the *At103* and ITS data for 23 samples. Insertions and deletions (indels) were coded as binary characters using the program SeqState [33] with the “simple coding” method [34]. The binary characters were combined with the nucleotide data using the program SequenceMatrix [35]. Bayesian inferences (BI) were conducted in MrBayes v.3.2.5 [36]. The best-fit nucleotide substitution models for ITS, and the exon and intron of *At103* were determined using the corrected Akaike information criterion (AICc) in jModelTest v.2.1.7, respectively [31]. In the Bayesian inference, two independent analyses starting from different random trees with three heated and one cold chain were run for 10,000,000 generations, and trees were sampled every 1,000 generations. 10,000 trees from each run were sampled in total. The first 2,500 trees from each run were discarded as burn-in, and the remaining 15,000 trees were used to construct a 50% majority-rule consensus tree and posterior probabilities (PP).

For the *s6pdh* data, boundaries of the exon2, intron2, exon3, intron3, exon4, intron4, exon5, intron5, and exon6 regions were determined by comparing with the published *s6pdh* sequence of *Prunus subcordata* Benth. [12]. Indel coding, selection of best-fitting nucleotide substitution models for each region, and Bayesian inference were performed as described above for *At103* and the *s6pdh*. Rapid bootstrap analysis was conducted with a random number seed and 1000 alternative runs using RAxML v.8.2 [37]. All tree visualizations were achieved with FigTree v1.4.2 (http://tree.bio.ed.ac.uk/software/figtree/).

We did not combine the *s6pdh* with the *At103* and ITS data because there were very few samples for which sequences from all three regions were available.

## Results

We isolated 212 sequences of the *At103* gene from 47 species of *Prunus* s.l. The length of the *At103* ranged from 444 bp to 538 bp. There were 228 variable characters, of which 136 (excluding indel sites) were parsimony-informative in the aligned matrix of 212 sequences. All the *At103* gene sequences contained the third exon and the intron between exons 3 and 4. The exon 3 region of the *At103* gene was conserved, consisting of 195 bp in the alignment without any indels. The length of the intron ranged from 249 to 343 bp. Modeltest indicated that the best-fit model under AICc was H80+G.

**Fig 2:**
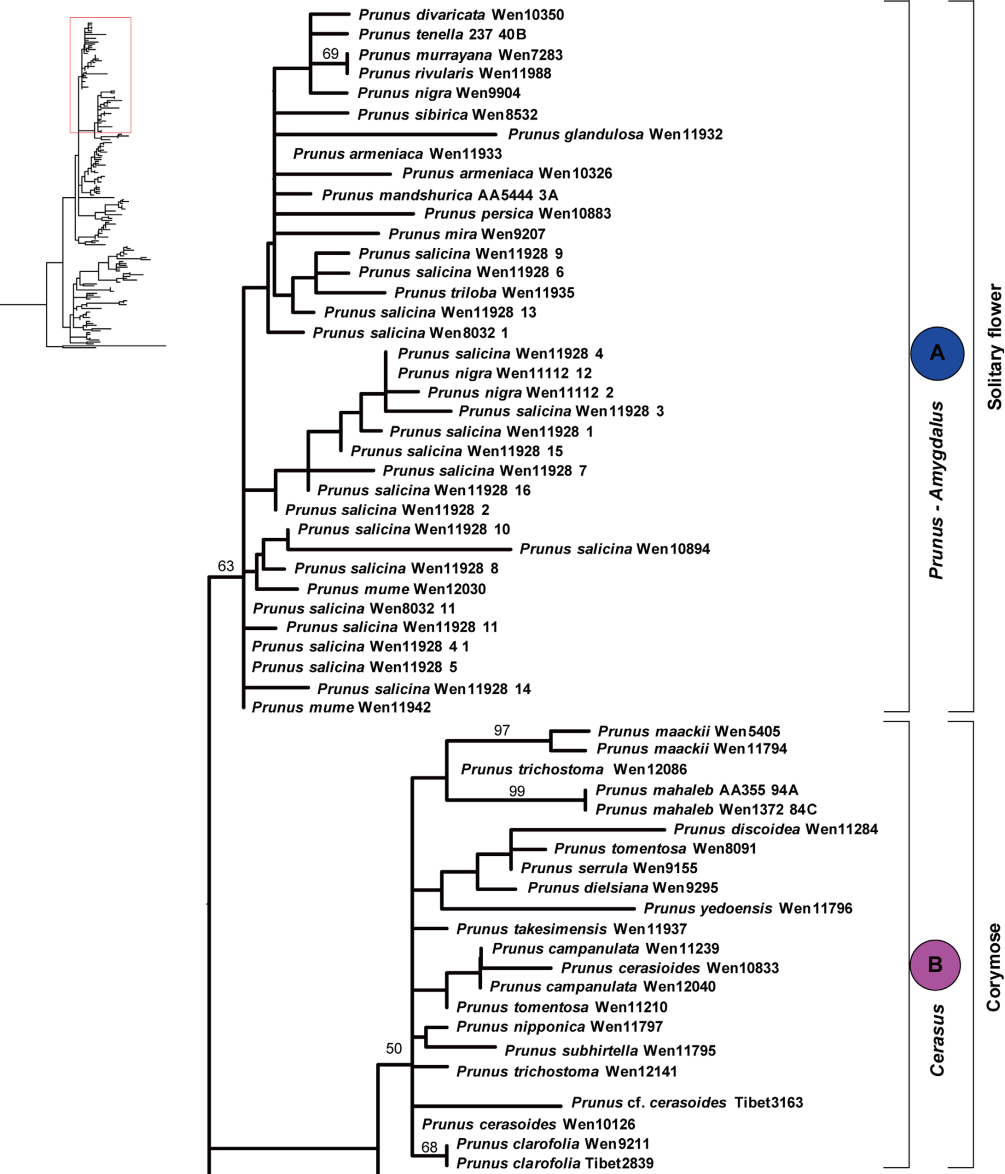
Maximum likelihood (ML) tree inferred from the *At103* DNA sequences of *Prunus*. The results of ML bootstrap analysis are shown above the branches. Bootstrap values >50% are shown.

**Fig 3:**
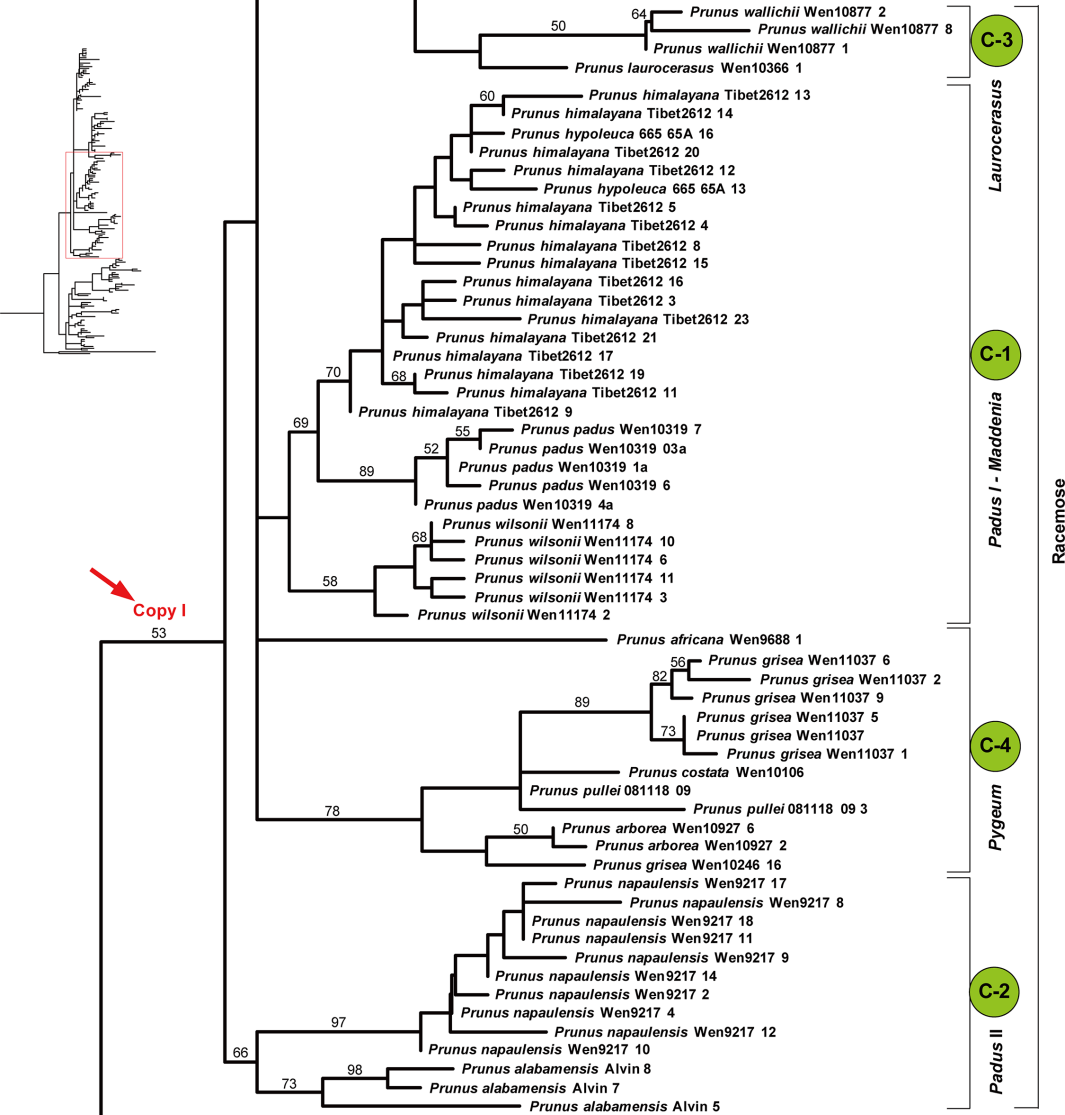
Maximum likelihood (ML) tree inferred from the *At103* DNA sequences of *Prunus*. The results of ML bootstrap analysis are shown above the branches. Bootstrap values >50% are shown.

**Fig 4:**
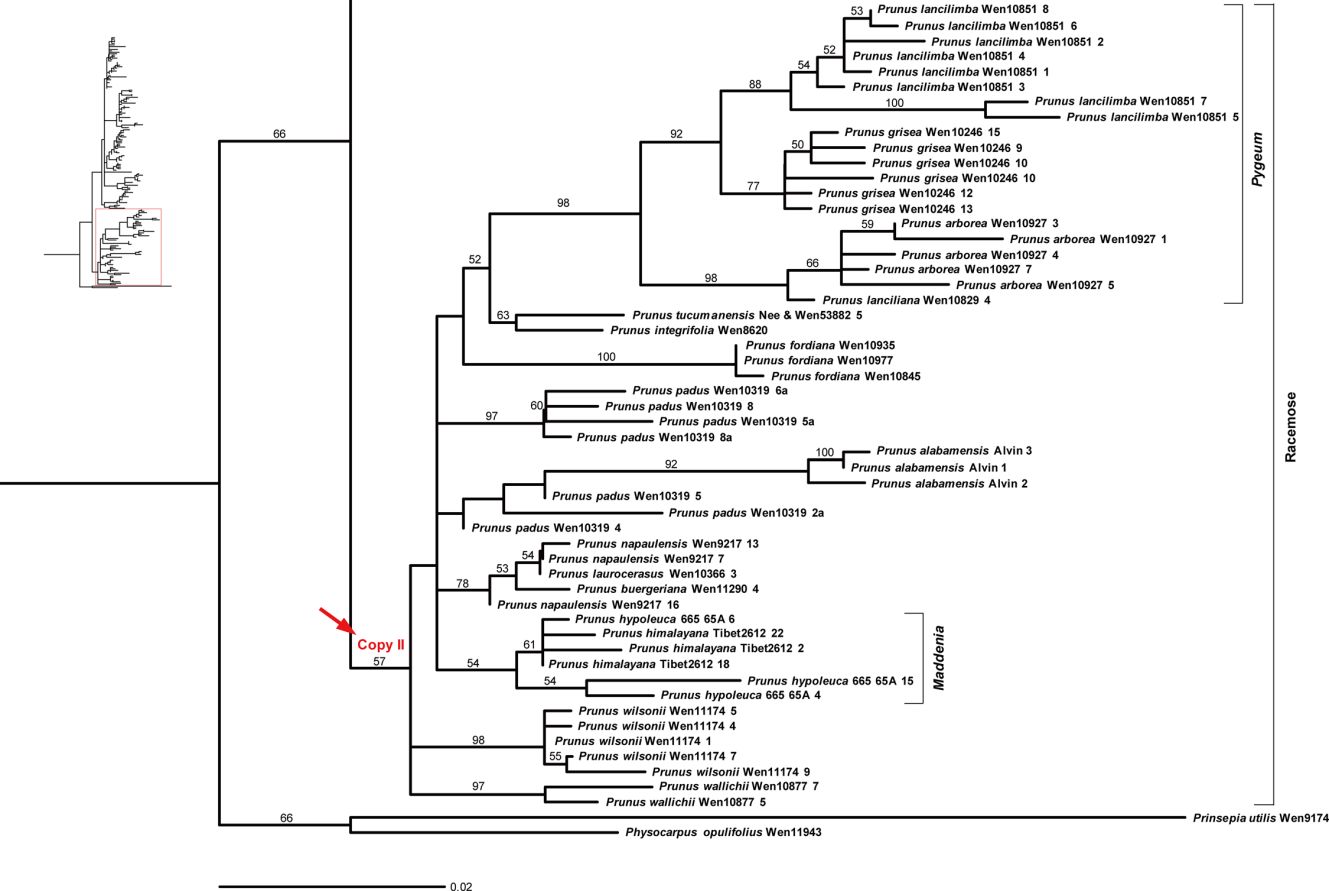
Maximum likelihood (ML) tree inferred from the *At103* DNA sequences of *Prunus*. The results of ML bootstrap analysis are shown above the branches. Bootstrap values >50% are shown.

The *At103* gene tree generated by maximum likelihood analyses with phyML suggested two major copies of the nuclear *At103* gene within *Prunus* s.l. (herein designated as copy I and copy II), but with weak support (Figs 2–4). Copy I was exhibited by 42 species whereas copy II was only found in 15 species, all belonging to the racemose group (Figs 2–4).

The length of copy I of the *At103* gene ranged from 458 to 538 bases. There were 155 variable characters, of which 77 were parsimony-informative in the aligned matrix of 118 sequences. Seven indels were present in the entire gene alignment. The indels consisted of 1–27 nucleotides. Three relatively large ones (a deletion of 27 bp, a deletion of 21 bp, and an insertion of 50 bp) were found in group A (*Prunus-Amygdalus*). The length of copy II of the *At103* gene ranged from 444 to 498 bases. There were 117 variable characters, of which 75 were parsimony-informative in the aligned matrix of 53 sequences. The alignment of the entire gene had six indels, each consisting of one to six nucleotides.

The copy I sequences supported the monophyly of the *Prunus-Amygdalus* group (group A), which possess solitary flowers. The ML tree also supported the *Cerasus* clade (group B), which has corymbose inflorescences. Sequences of the racemose species did not form a monophyletic group, and four subgroups may be identified, and defined by morphology and geography. Subgroups C-1 and C-2 include the species from temperate zone (*Padus* I-*Maddenia* and *Padus* II). Species formerly classified in *Maddenia* were nested within subgenus *Padus*. Subgroup C-3 includes the European species *P*. *laurocerasus* and the subtropical and tropical Asian species *P*. *wallichii* of subgenus *Laurocerasus*. Subgroup C-4 consists of the tropical species from Southeast Asia belonging to the *Pygeum* group of subgenus *Laurocerasus* and the African species *Prunus africana*.

The copy II sequences were only found in species of the racemose group. The sequences supported the monophyly of the *Pygeum* group. Also, the Neotropical *Prunus integrifolia* and *P*. *tucumanensis* formed another clade. The *Pygeum* group was shown to be sister to this Neotropical clade, but with low bootstrap support. Species formerly assigned to *Maddenia* formed a clade. Other relationships within the racemose group were poorly resolved based on the copy II sequences.

A neighbornet diagram (S1 Fig) suggested two major splits, corresponding to copy I and copy II of the *At103* sequences. The copy I sequences can distinguish three broad groups: group A (corresponding to the *Prunus-Amygdalus* group in Figs 2–4), group B (corresponding to *Cerasus* group in Fig 2) and group C. Group C comprised species of the racemose group with four subgroups supported by copy I sequences, i.e., C-1, C-2, C-3 and C-4 (roughly corresponding to subgroups *Padus* I-*Maddenia*, *Padus* II, *Laurocerasus*, and *Pygeum* in Figs 2–4). Copy II was only possessed by species of the racemose group and it did not provide strong resolution of relationships within the group, although species of the *Pygeum* group were supported to form a cluster (S1 Fig).

**Fig 5:**
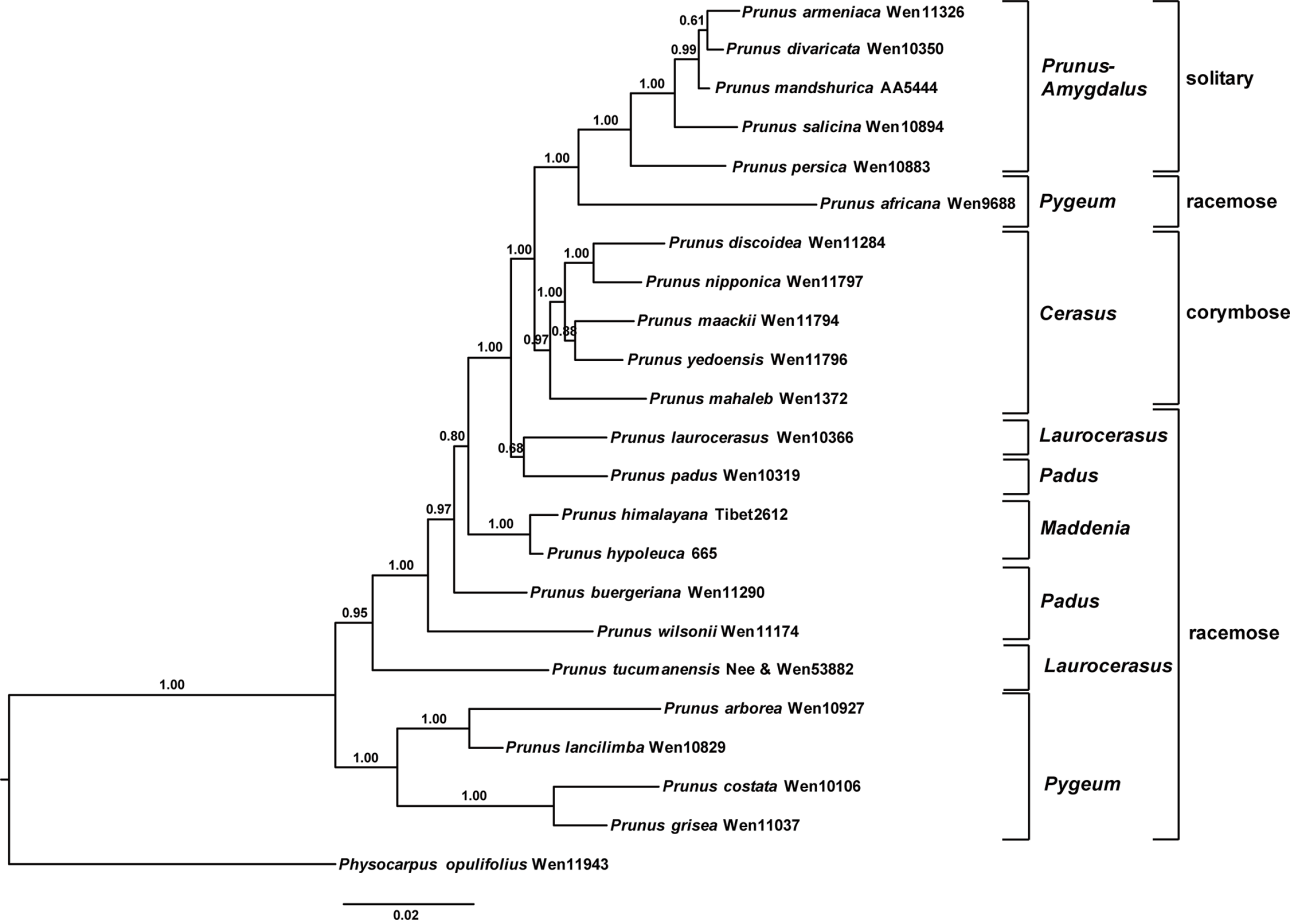
The 50% majority-rule consensus tree of Bayesian analysis inferred from the combined *At103* and ITS sequences of *Prunus*. Bayesian posterior probabilities are shown above the branches.

The combined *At103*-ITS data set had 1136 characters, of which 283 were variable and 134 were parsimony-informative in the aligned matrix of 23 sequences. Modeltest indicated that under AICc, the best-fit models for ITS and the exon and intron of *At103* were TIM2+I+G, K80 and TPM2uf, respectively. The combined *At103* and ITS sequences supported the monophyly of the *Prunus* s.s.*- Amygdalus* group (PP = 1.00). The Bayesian tree also supported the *Cerasus* clade (PP = 0.97). Sequences of the racemose species were resolved as paraphyletic (Fig 5). Species formerly classified in *Maddenia* formed a clade (PP = 1.00). The *Pygeum* group formed a clade, with the exception of the only African member of the group, *P*. *africana*, which was resolved as sister to the *Prunus* s.s.—*Amygdalus* clade. Species of subgenera *Padus* and *Laurocerasus* were highly mixed with each other (Fig 5).

Thirty-eight sequences of *s6pdh* gene were isolated from 26 species of *Prunus* s.l. The length of the *s6pdh* sequences ranged from 1163 bp to 1335 bp. The aligned data set of 38 sequences had 1377 characters, of which 653 were variable and 318 (excluding indel sites) were parsimony-informative. The exon regions of the *s6pdh* gene were conserved relatively. The length of introns ranged from 125 to 187 bp. Modeltest indicated that the best-fit models under AICc for exon2, intron2, exon3, intron3, exon4, intron4, exon5, intron5, and exon6 were JC, JC, JC, HKY+G, K80+G, TPM3, K80+G, K80, TPM3+G, respectively.

**Fig 6:**
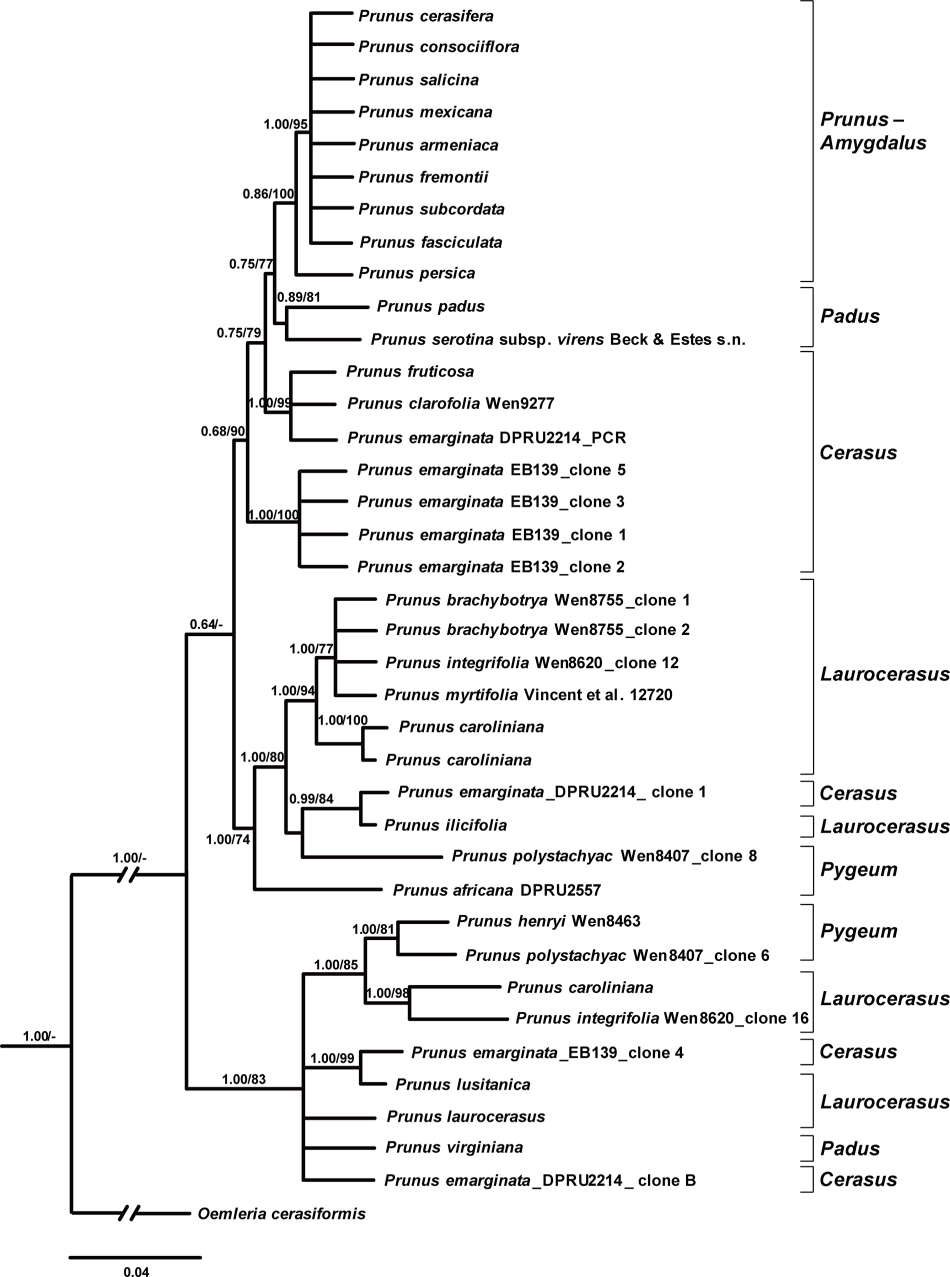
Bayesian tree inferred from the *s6pdh* DNA sequences of *Prunus*. Bayesian posterior probabilities (left) (≥ 0.95) and likelihood bootstrap (right) values (≥ 50%) are given above branches. Dashes represent bootstrap ≤ 50%.

Phylogenetic analyses of the *s6pdh* sequences supported the monophyly of the *Prunus-Amygdalus* group, whose members bear solitary flowers (PP = 1.00, BS = 95%). Sequences of the *Cerasus* species did not form a monophyletic group and were nested within racemose group (*Padus-Laurocerasus-Pygeum*) (Fig 6).

The relationships of *s6pdh* sequences of *Prunus emarginata* were complex. The sequences from the accession EB139 were grouped in two separate clades, with one clone (#4) grouping with *P*. *lusitanica* of the *Laurocerasus* group, and the other four clones (#1–3, & 5) forming a clade sister to the main *Prunus-Amygdalus-Cerasus* group plus *P*. *padus*–*P*. *serotina* subsp. *virens *(Fig 6). The sequences of *P*. *emarginata* from the second accession (DPRU2214) were shown in three different clades, which were scattered in the *Cersaus* and *Laurocerasus*-*Padus* groups (sister to *Prunus ilicifolia* of the *Laurocerasus* group; sister to a large clade of the *Laurocerasus-Padus-Pygeum* group; or sister to the *P*. *fruticosa–P*. *clarofolia* of the *Cerasus* group).

## Discussion

Two copies of *At103* gene were detected in the species of the polyploid racemose group in *Prunus*. The topologies of the *At103*, the combined *At103*-ITS data, and the *s6pdh* data are generally similar to each other, but clearly different from that of the plastid tree (cf. Figs 1–6 and S1 Fig). The incongruent relationships in the polyploid racemose group in *Prunus*, as also observed in the separate phylogenetic analyses of plastid and nuclear ITS sequences in previous studies [3, 4, 10, 13, 15], have been hypothesized to be the result of an ancient hybridization event [4].

Chromosome numbers provide further evidence for the possible hybrid origin of the racemose group. The base chromosome number of *Prunus* is *x* = 8. Most of the species in the solitary flower group (e.g., peach, *P*. *persica*; almond, *P*. *dulci*) and the corymbose group (e.g., sweet cherry, *P*. *avium*) have the chromosome number of 2*n* = 2*x* = 16. On the other hand, species from the racemose group have been reported to have higher ploidy levels (e.g., 2*n* = 4*x* = 32 for most species; *P*. *lusitanica*, 2*n* = 8*x* = 64, and *P*. *laurocerasus*, 2*n* = 22*x* = 176) [18]. The higher ploidy levels of these species indicate that polyploidization may have played a role in the origin(s) of the entire racemose group.

It is well documented that hybrid-mediated genome doubling (allopolyploidy) has played an important role in plant evolution [4, 38–41]. Speciation involving allopolyploidy may have occurred repeatedly in different geographic locations and at different times, which may result in morphological differences between hybrids of the same parentage [42].

In the previously generated nuclear ITS tree, the racemose group was resolved as paraphyletic [3–5, 11]. The taxa in the racemose group were also not supported to be monophyletic by the *At103*, *At103* and ITS, and *s6pdh* trees (Figs 2–6 and S1 Fig). and these taxa did not form a cluster in the neighbornet diagram (S1 Fig). Four subgroups were resolved within the racemose group by copy I of the *At103* gene data, corresponding to: (1) the temperate subgenus *Padus* (I) and former genus *Maddenia*; (2) the temperate subgenus *Padus* (II); (3) the European and the subtropical Asian members of subgenus *Laurocerasus* and (4) the *Pygeum* group from Southeast Asia, Africa and Australia (part of subgenus *Laurocerasus*) (Figs 2–4 and S1 Fig). The three to four lineages to a large extent have morphological and geographic integrity. Both subgenus *Padus* and the former genus *Maddenia* are deciduous and distributed in temperate regions. The taxa of subgenus *Laurocerasus* are evergreen with axillary inflorescences that are leafless at the base of the rachis, and are distributed in tropical and subtropical regions of both the New and Old Worlds. The *Pygeum* group of *Laurocerasus* is further characterized by indistinguishable sepals and petals, and is distributed mainly in tropical Asia and Africa with one species in Australia [16].

The phylogenetic trees based on the combined *At103*-ITS (Fig 5), and *s6pdh* (Fig 6) data are largely congruent with the trees based on separate analyses of ITS or *At103* [4, 5, 10]. The *Prunus-Amygdalus* and the *Cerasus* groups are nested within a paraphyletic racemose group (*Padus-Laurocerasus-Pygeum*) (Figs 5 and 6). In the *s6pdh* tree, the taxa of the *Cerasus* group did not form a monophyletic group, with each individual of *Prunus emarginata* showing at least two copies (Fig 6).

In most angiosperms, the plastid genome is maternally inherited while the nuclear genome is biparentally inherited [43]. Therefore, the maternal and paternal parent(s) that contributed to the hybrid origin of the racemose group may be inferred by comparing the results of phylogenetic analyses of the plastid DNA [4] with those from the nuclear *At103*, ITS and *s6pdh* DNA sequences. Our data support the hypothesis that allopolyploidy was involved in the origin of the racemose lineages of *Prunus*, as previously suggested, and further suggest that several independent allopolyploidy events occurred.

The maternal parent(s) of the racemose group must have belonged to an early-diverging lineage of *Prunus*, as plastid data support three major clades in the genus and resolve the *Laurocerasus-Padus-Maddenia* clade (the racemose group) as sister to a clade including the *Prunus-Amygdalus* clade (the solitary flower group) plus the *Cerasus* clade (the corymbose groups) [3, 4]. The maternal lineage(s) may have been an extinct widespread species or several species belonging to the same lineage of group C in the *At103* gene tree topology.

**Fig 7:**
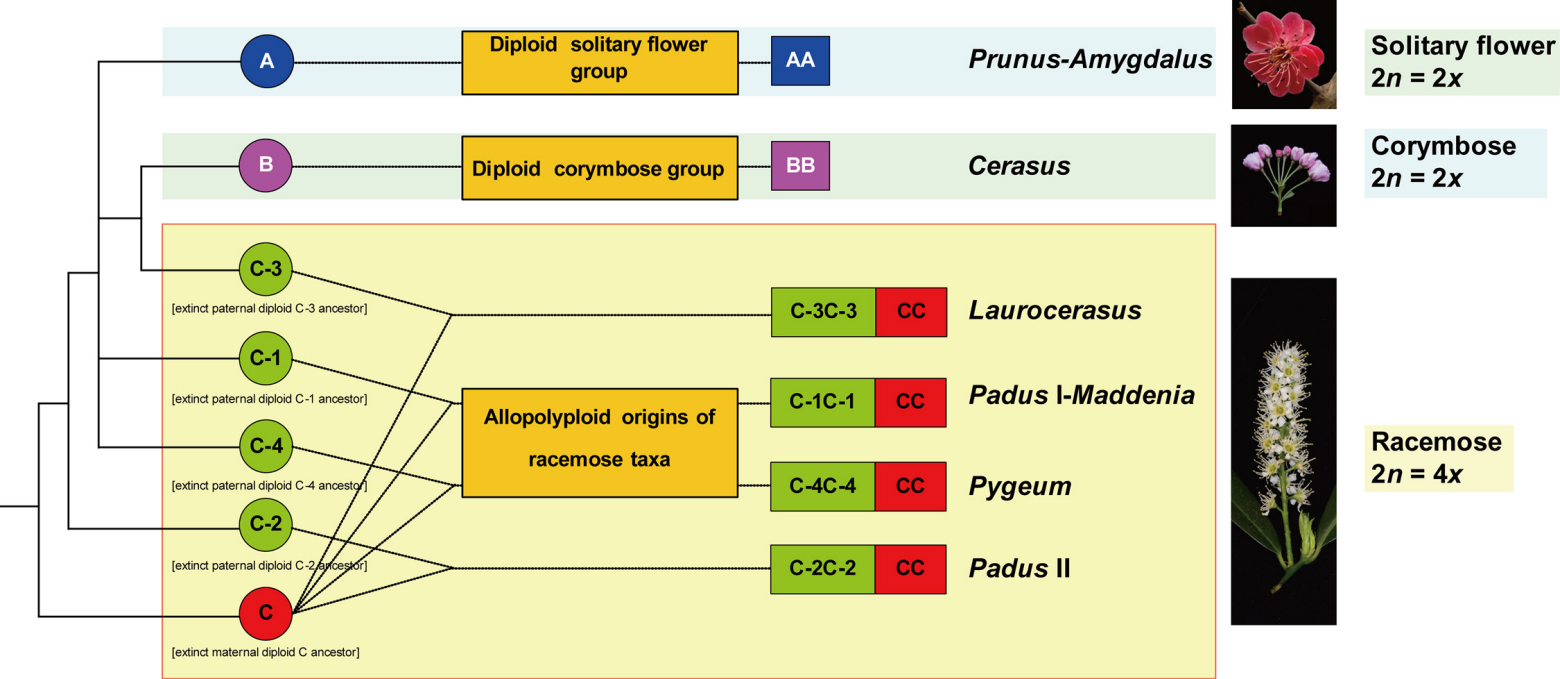
Hypothesized evolutionary history of *Prunus*, highlighting independent allopolyploidy events in subgroup *Padus* I*-Maddenia* (C-1C-1CC), *Padus* II (C-2C-2CC), *Laurocerasus* (C-3C-3CC), and *Pygeum* (C-4C-4CC). Photographs (top to bottom): *Prunus mume*; *P*. *yedoensis*; *P*. *laurocerasus*. A = solitary flower group; B = corymbose inflorescence group; C = racemose inflorescence group.

In contrast, our data suggest that the paternal parents involved in the multiple allopolyploidy events that gave rise to the racemose lineages of *Prunus* were more diverged. The *At103* phylogeny suggests that some lineages have retained the paternal copy (subgroup C-1, C-2, C-3, C-4), while others have retained the maternal copy (group C in copyII). Collectively, these four subgroups (C-1, C-2, C-3, and C-4) of the racemose group in copy I and the group C in copy II reveal the paternal and maternal ancestral genome donors for the racemose group in *Prunus*, respectively (Fig 7). Patterns of molecular phylogenetic topologies from the nuclear *At103*, ITS and *s6pdh* and the chloroplast genome and the non-random morphological variations best support the hypothesis of independent events of allopolyploidy in taxa within the racemose group.

In their recent classification of *Prunus*, Shi et al. [7] proposed that taxa of the racemose group should be treated as only one subgenus *Padus*. Our hypothesis of independent events of allopolyploidy in taxa within the racemose group argues against recognizing all species of the group as one subgenus (Fig 7). The species with racemose inflorescences may still need to be treated taxonomically as belonging to several subgenera based on both morphology and the nuclear sequence data.

The time of the first formation of the racemose group was estimated to be 55.4 (45.1–66.3) Myr [4]. Divergence times for subgroups C-1, C-2, C-3, and C-4 of the racemose group were estimated from 37.2 to 14.9 Myr, at different times [4]. Thus the multiple hybridization events may have happened at different times. Furthermore, these multiple allopolyploidy events may have also occurred in different regions, e.g., temperate zone for subgroup C-1 and C-2; the European and subtropical Asian region for subgroup C-3, and Southeast Asian, African tropics and Australia tropics for subgroup C-4. However, the events may have happened so long ago that the diploid ancestral taxa have become extinct, and no extant diploid representatives of the racemose group are known.

The *Maddenia* group was previously shown to be closely related to subgenera *Laurocerasus* and/or *Padus* by phylogenetic studies [3–5, 13, 15]. The *At103* gene sequences showed that *Maddenia* was nested within a subgroup composed of some members of *Padus* (I) (*Prunus padus* and *P*. *wilsonii*) in copy I, with other species of *Padus* (II) constituting another subgroup (Fig 5), which is consistent with the phylogenetic results based on sequences of ITS, *ndhF*, *rps16* and *rpl16* [11]. The combined *At103*-ITS sequences also showed that *Maddenia* was nested within subgroups *Padus* and *Laurocerasus*.

Members of the *Pygeum* group have a perianth without differentiated petals [17]. This group has been shown to be nested within the *Laurocerasus-Padus* complex based on nuclear and plastid sequences [3, 11]. The *At103* neighbornet diagram and the combined *At103*-ITS data both suggest that species of *Pygeum* formed a group; however, the phylogenetic position of the African species *Prunus africana* (also formerly classified in *Pygeum*) still remains controversial (Figs 2–5 and S1 Fig). *Prunus africana* possesses some unique characters, such as leaves with incised margins and the glands situated in the margin, but distinct from other taxa of *Pygeum*. Its position needs to be explored further in future analyses.

Allopolyploidy in *Prunus* resulting from the fertilization of unreduced female gametes has been reported between diploid and tetraploid species with the evidence for gametophytic apomixis in the genus [44]. Future work on the genus needs to investigate this aspect of *Prunus* reproductive biology to gain insights into the mechanisms of allopolyploidy.

*Prunus emarginata* has been treated as a member of subgenus *Cerasus* [2]. The *s6pdh* sequence data suggest a highly complex pattern in the species (Fig 6). Our sequences were from two individuals (specimen vouchers: EB139 and DPRU 2214). Each individual has at least two copies of the *s6pdh* gene, suggesting that hybridization may have been involved in the origin of the species. Individuals of *P*. *emarginata* vary considerably in the habit, size and shapes of leaves and inflorescences. Its inflorescence is intermediate between that of the *Cerasus* group and the *Padus* group. The *s6pdh* sequences also place it either with the *Prunus-Amygdalus-Cerasus* group or with the racemose group. Fertile hybrids between *P*. *emarginata* and naturalized *P*. *avium* [45], *P*. *emarginata* and *P*. *pensylvanica* [46] have been reported. Clearly our *s6pdh* data support a highly complex genetic profile of these species involving reticulate evolution. Unfortunately, the chromosome number of the species is unknown and should be studied.

In conclusion, the hypothesis of multiple events of allopolyploidy in the evolution of the racemose lineages in *Prunus* is supported by our combined evidence from nuclear and plastid markers. A widespread early diverged lineage of *Prunus* is suggested to have served as the maternal parent(s) for multiple allopolyploidy events involving several paternal lineages. This hypothesis of the evolutionary history of the racemose group in *Prunus* reflects a major step forward in our understanding of *Prunus* diversification. Further analyses using more nuclear DNA sequences via next-generation sequencing [47, 48] are needed to produce a robust nuclear phylogeny for the interpretation of the evolutionary diversification of this economically important genus.

## Supporting Information

S1 Fig Neighbornet diagram based on uncorrected *P* distances of nuclear *At103* DNA sequences of *Prunus*. The dash lines indicate the discrimination of two potential copies of *At103* gene. The solid lines indicate seven major lineages of *Prunus*. The red and black numbers indicate the species and bootstrap support values, respectively. Each species is designated with a number as follows: 1. *P*. *armeniaca*; 2. *P*. *divaricata*; 3. *P*. *glandulosa*; 4. *P*. *mandshurica*; 5. *P*. *mume*; 6. *P*. *salicina*; 7. *P*. *sibirica*; 8. *P*. *murrayana*; 9. *P*. *rivularis*; 10. *P*. *nigra*; 11. *P*. *mira*; 12. *P*. *persica*; 13. *P*. *triloba*; 14. *P*. *tenella*; 15. *P*. *tomentosa*; 16. *P*. *trichostoma*; 17. *P*. *serrula*; 18. *P*. *dielsiana*; 19. *P*. *cerasoides*; 20. *P*. *trichostoma*; 21. *P*. *campanulata*; 22. *P*. *clarofolia*; 23. *P*. *discoidea*; 24. *P*. *maackii*; 25. *P*. *mahaleb*; 26. *P*. *nipponica*; 27. *P*. *subhirtella*; 28. *P*. *takesimensis*; 29. *P*. *yedoensis*; 30. *P*. *padus*; 31. *P*. *wilsonii*; 32. *P*. *himalayana*; 33. *P*. *hypoleuca*; 34. *P*. *alabamensis*; 35. *P*. *napaulensis*; 36. *P*. *wallichii*; 37. *P*. *laurocerasus*; 38. *P*. *africana*; 39. *P*. *arborea*; 40. *P*. *costata*; 41. *P*. *grisea*; 42. *P*. *pullei*; 43. *P*. *buergeriana*; 44. *P*. *integrifolia*; 45. *P*. *fordiana*; 46. *P*. *lancilimba*; 47. *P*. *tucumanensis*; 48. *Physocarpus opulifolius*; 49. *Prinsepia utilis*.
(TIF)
