# Supplementary figures and images for: Multiple Events of Allopolyploidy in the Evolution of the Racemose Lineages in Prunus (Rosaceae) Based on Integrated Evidence from Nuclear and Plastid Data
Source: PLoS One. 2016 Jun 13;11(6):e0157123. doi: 10.1371/journal.pone.0157123 (PMC4905661; doi:10.1371/journal.pone.0157123)

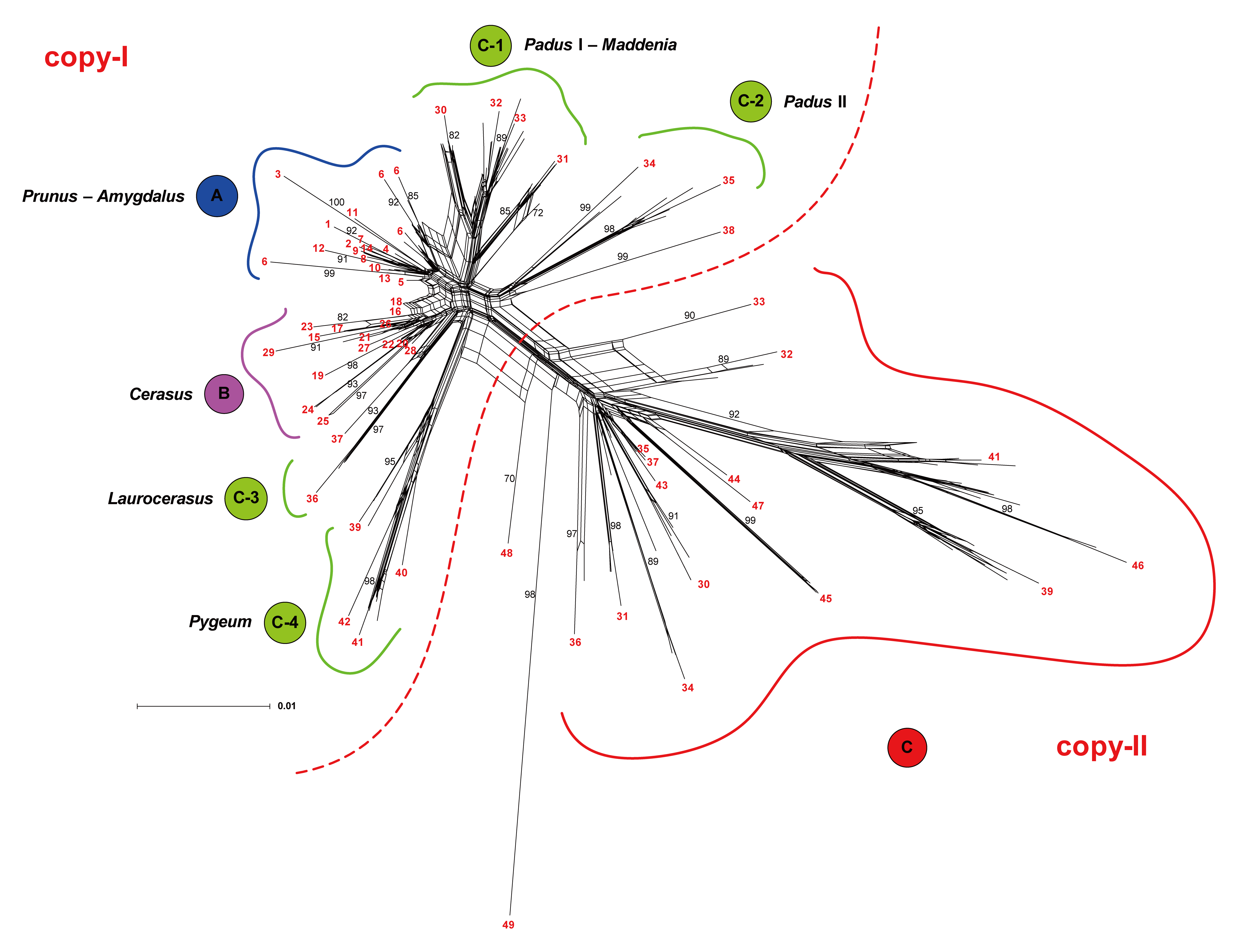

Supplement: S1 Fig — The dash lines indicate the discrimination of two potential copies of At103 gene. The solid lines indicate seven major lineages of Prunus. The red and black numbers indicate the species and bootstrap support values, respectively. Each species is designated with a number as follows: 1. P. armeniaca; 2. P. divaricata; 3. P. glandulosa; 4. P. mandshurica; 5. P. mume; 6. P. salicina; 7. P. sibirica; 8. P. murrayana; 9. P. rivularis; 10. P. nigra; 11. P. mira; 12. P. persica; 13. P. triloba; 14. P. tenella; 15. P. tomentosa; 16. P. trichostoma; 17. P. serrula; 18. P. dielsiana; 19. P. cerasoides; 20. P. trichostoma; 21. P. campanulata; 22. P. clarofolia; 23. P. discoidea; 24. P. maackii; 25. P. mahaleb; 26. P. nipponica; 27. P. subhirtella; 28. P. takesimensis; 29. P. yedoensis; 30. P. padus; 31. P. wilsonii; 32. P. himalayana; 33. P. hypoleuca; 34. P. alabamensis; 35. P. napaulensis; 36. P. wallichii; 37. P. laurocerasus; 38. P. africana; 39. P. arborea; 40. P. costata; 41. P. grisea; 42. P. pullei; 43. P. buergeriana; 44. P. integrifolia; 45. P. fordiana; 46. P. lancilimba; 47. P. tucumanensis; 48. Physocarpus opulifolius; 49. Prinsepia utilis. (TIF) [file pone.0157123.s001.tif]
